# Durvalumab following definitive chemoradiotherapy among patients with stage III NSCLC in the Thai population: a real-world, multicenter observational study

**DOI:** 10.3389/fonc.2025.1647385

**Published:** 2025-10-22

**Authors:** Piyada Sitthideatphaiboon, Thanyanan Reungwetwattana, Sureerat Jaruhathai, Archara Supavavej, Piyarat Limpawittayakul, Kunlatida Maneenil, Krittiya Korphaisarn, Thatthan Suksombooncharoen, Luangyot Thongthieang, Chirawadee Sathitruangsak, Chawalit Chayangsu, Hataiwan Ratanabunjerdkul, Naiyarat Prasongsook, Virote Sriuranpong

**Affiliations:** ^1^ Division of Medical Oncology, Department of Medicine, Faculty of Medicine, Chulalongkorn University and King Chulalongkorn Memorial Hospital, Bangkok, Thailand; ^2^ Division of Medical Oncology, Department of Medicine, Faculty of Medicine, Ramathibodi Hospital, Mahidol University, Bangkok, Thailand; ^3^ Department of Internal Medicine, Police General Hospital, Bangkok, Thailand; ^4^ Division of Medical Oncology, Chulabhorn Hospital, Chulabhorn Royal Academy, Bangkok, Thailand; ^5^ Oncology Unit, Department of Medicine, Rajavithi Hospital, College of Medicine, Rangsit University, Bangkok, Thailand; ^6^ Division of Medical Oncology, Department of Medicine, Faculty of Medicine, Siriraj Hospital, Mahidol University, Bangkok, Thailand; ^7^ Division of Oncology, Department of Internal Medicine, Faculty of Medicine, Chiang Mai University, Chiang Mai, Thailand; ^8^ Department of Medicine, Medical Oncology Unit, Department internal Medicine, Khon Kaen Hospital, Khon Kaen, Thailand; ^9^ Holistic Center for Cancer Study and Care (HOCC-PSU) and Department of Medicine, Faculty of Medicine, Prince of Songkla University, Songkhla, Thailand; ^10^ Department of Internal Medicine, Surin Hospital, Institute of Medicine, Suranaree University of Technology, Surin, Thailand; ^11^ Medical Oncology Unit, Department of Internal Medicine, Thammasat University, Pathum Thani, Thailand; ^12^ Division of Medical Oncology, Department of Medicine, Phramongkutklao Hospital and College of Medicine, Bangkok, Thailand

**Keywords:** locally advanced (stage III) non-small cell lung cancer, durvalumab, immunothearpy, consolidation therapy, real world data (RWD)

## Abstract

**Background:**

In locally advanced non-small cell lung cancer (LA-NSCLC), durvalumab as consolidation therapy following definitive chemoradiotherapy (CRT) was established as the standard of care. Given the heterogeneity of patients with LA-NSCLC, the present study evaluated the efficacy and safety of durvalumab in a real-world, multicenter observational study.

**Methods:**

Patients with LA-NSCLC, whose disease had not progressed following CRT and receiving ≥1 dose of durvalumab as part of the expanded access program (EAP) in Thailand and outside EAP, were included. In addition to descriptive statistics, survival probability was determined using the Kaplan–Meier method.

**Results:**

A total of 82 patients from 12 centers in Thailand were enrolled. The median age was 63 years, 74% were men, 72% had non-squamous NSCLC, and 20% of patients had an epidermal growth factor receptor (EGFR) mutation. Only 13.4% of patients were tested for programmed death-ligand 1 (PD-L1), and 54.5% had PD-L1 expression. Most patients (84%) received concurrent CRT, and carboplatin/paclitaxel was the most commonly used. Of the patients, 89% received radiotherapy (RT) dose ≥60 Gy with a median time of durvalumab initiation from the end of RT being 42 days. Overall, 57% of patients completed the 12-month treatment with a median of 24 cycles. Objective response rate (ORR) and disease control rate (DCR) were 41.3% and 86.7%, respectively. With a median follow-up time of 43.3 months, 2-year progression-free survival (PFS) and 2-year time to second objective disease progression (PFS2) were 63.1% and 81.6%, respectively. Immune-related adverse events (irAEs) of any grade and grade ≥ 3 were 25.6% and 9.8%, respectively. Pneumonitis was the most frequent irAE (17%), and 6% were grade ≥ 3, leading to discontinuation in six patients (7.3%).

**Conclusions:**

Durvalumab following definitive CRT demonstrated promising outcomes and was well-tolerated in this real-world study. These findings support the utilization of durvalumab for enhancing outcomes in patients with unresectable LA-NSCLC within Asian populations.

## Background

Lung cancer is the most common cause of cancer-related mortality worldwide and poses a major health problem throughout the world (1). In Thailand, lung cancer is the second and fourth most common cancer among men and women, respectively. Non-small cell lung cancer (NSCLC) accounts for almost 85% of all lung cancer cases (2), and approximately one-third of patients are diagnosed with stage III, locally advanced (LA) disease (3). The standard of care for patients with a good performance status and unresectable stage III NSCLC used to be platinum-based doublet chemotherapy concurrent with radiotherapy [chemoradiotherapy (CRT)]. However, the outcomes of treatment for patients with LA-NSCLC remain poor, with a median progression-free survival (PFS) of approximately 8 months, and only 15% of patients are alive at 5 years (4). Major advances in the treatment of patients in this context have been made in recent years. One such advance is durvalumab, which is a human monoclonal antibody that blocks the interaction of programmed cell death-ligand 1 (PD-L1) with programmed cell death protein 1 (PD-1) and a cluster of differentiation 80 (CD80) by binding to PD-L1. In the phase 3 PACIFIC trial, durvalumab was found to significantly improve PFS in stage III NSCLC patients when given as consolidation therapy following definitive CRT (5), and more recently, it has been announced that the drug also significantly improved overall survival (OS) (6). Based on these findings of the PACIFIC trial, consolidation durvalumab after CRT was further established as the new standard of care in this setting (7).

Patients with stage III NSCLC represent a very heterogeneous group with diverse tumor and nodal statuses, and the therapeutic approach may vary widely across countries (8). Real-world evidence, the collection of data from daily medical practice with less-restrictive eligibility criteria than a clinical trial, played an increasing role in understanding these diverse practice patterns and treatment pathways, which may gain disease insights and enable clinicians to make optimum clinical judgments (9). PACIFIC Real-World (PACIFIC-R) study, an international, observational study that enrolled patients who have received durvalumab as part of expanded access programs (EAPs) in European countries, provided the first real-world data on the use and effectiveness of the PACIFIC regimen (10). However, data were collected in the PACIFIC-R study largely in European populations in which the prevalence of epidermal growth factor receptor (*EGFR*) mutation is much lower than that in Asian populations. The benefit of consolidation durvalumab is still uncertain in patients with *EGFR* mutations, as they constituted only 6% and 7.9% in the PACIFIC and PACIFIC-R studies, respectively (5, 10). Here, we reported the multicenter, observational, retrospective study that explored the treatment effectiveness outcomes and safety of durvalumab as consolidation therapy following definitive CRT in patients with LA-NSCLC in the real-world setting, especially in Asian populations.

## Methods

### Study population

A multicenter, observational, retrospective study was conducted on patients with unresectable stage III NSCLC who were treated with definitive chemoradiation and were eligible for consolidation therapy with durvalumab, either as part of the EAP in Thailand or outside the EAP. A total of 82 patients from 12 centers were enrolled between January 1, 2021, and August 31, 2022. Data for this study were extracted from patients’ medical records and entered into electronic case report forms (eCRFs) within the Thailand National Lung Cancer Registry Database.

### Statistical analysis

Continuous and ordinal variables were summarized as median and range, and categorical variables were reported as counts and percentages. The relationship between variables was assessed using Pearson’s chi-square test or Fisher’s exact test as appropriate. Outcomes were analyzed in terms of PFS, time to second objective disease progression (PFS2), objective response rate (ORR), disease control rate (DCR), duration of response (DoR), and OS.

The index date was defined as the date of diagnosis. PFS was calculated from the index date to the first documented disease recurrence or death from any cause. PFS2 was calculated from the index date to objective progression on subsequent therapy or death. ORR was expressed as the number and percentage of patients with the best response following the Response Evaluation Criteria in Solid Tumors (RECIST) criteria, as available in the medical record. DoR was measured from the date of documented tumor response to the first recurrence. OS was measured from the index date to death from any cause. Concurrent chemoradiation was defined in accordance with the PACIFIC criteria as the administration of two or more cycles of platinum-based chemotherapy given concurrently with definitive radiotherapy. Sequential chemoradiation was defined as at least two cycles of platinum-based chemotherapy followed by radiotherapy, with a one-cycle overlap between chemotherapy and radiotherapy permitted. Time from the end of radiation to durvalumab initiation was calculated as the sum of days from the last radiation dose to randomization plus days from randomization to the first durvalumab dose, which was consistent with the PACIFIC study (the PACIFIC study protocol allowed 1–42 days from the last radiation dose to randomization). Patients who did not develop the event at the end of the study were censored at the date of the last observation, which was defined as December 31, 2022. The survival probability was computed using the Kaplan–Meier method, and heterogeneity in survival rates among strata was assessed using the log-rank test. Univariate and multivariate analyses were performed using the Cox model, and hazard ratios (HRs) and 95% confidence intervals (95% CIs) were calculated. *p*-Values <0.05 were considered statistically significant.

Additionally, a *post-hoc* analysis was also calculated using the initiation date of durvalumab as the index date, consistent with the PACIFIC and PACIFIC-R studies. Survival endpoints were calculated from the date of diagnosis as the primary analysis, with durvalumab initiation as the secondary analysis for comparison with published studies. Median follow-up time was estimated using the reverse Kaplan–Meier method, with the index date defined as the date of diagnosis. All the above statistical analyses were conducted using GraphPad Prism version 8.00 for Windows (GraphPad Software, La Jolla, CA, USA) and SPSS 23.0 (SPSS Inc., Chicago, IL, USA).

*Post-hoc* power calculations were performed to assess the adequacy of our sample size for detecting clinically meaningful differences in the primary endpoint of PFS using G*Power 3.1.9.7 and the R package “powerSurvEpi”. Our sample size was 82 patients, and the observed median PFS was 40.1 months (from durvalumab initiation). Assumed clinically meaningful hazard ratio was 0.7, alpha level was 0.05 (two-sided), and expected event rate based on observed was 46.3% progression events; therefore, the calculated power was 85.2% for detecting HR ≤ 0.7.

### Study period

The study protocol defined enrollment criteria whereby patients previously participating in the EAP were transitioned to the study following EAP discontinuation in Thailand on December 31, 2020 (ESR-18-14304_ESR Full Protocol, **Supplementary Material**). The formal study enrollment period commenced on January 1, 2021, and concluded on August 31, 2022, representing a 20-month recruitment window.

Follow-up duration was assessed using the reverse Kaplan–Meier method, which treated clinical events (death and disease progression) as censored observations rather than endpoints. This statistical approach provided an unbiased estimation of follow-up adequacy by accounting for patients whose observation period was terminated by clinical events rather than administrative study closure.

## Results

### Patient characteristics of the study population

**Figure 1:**
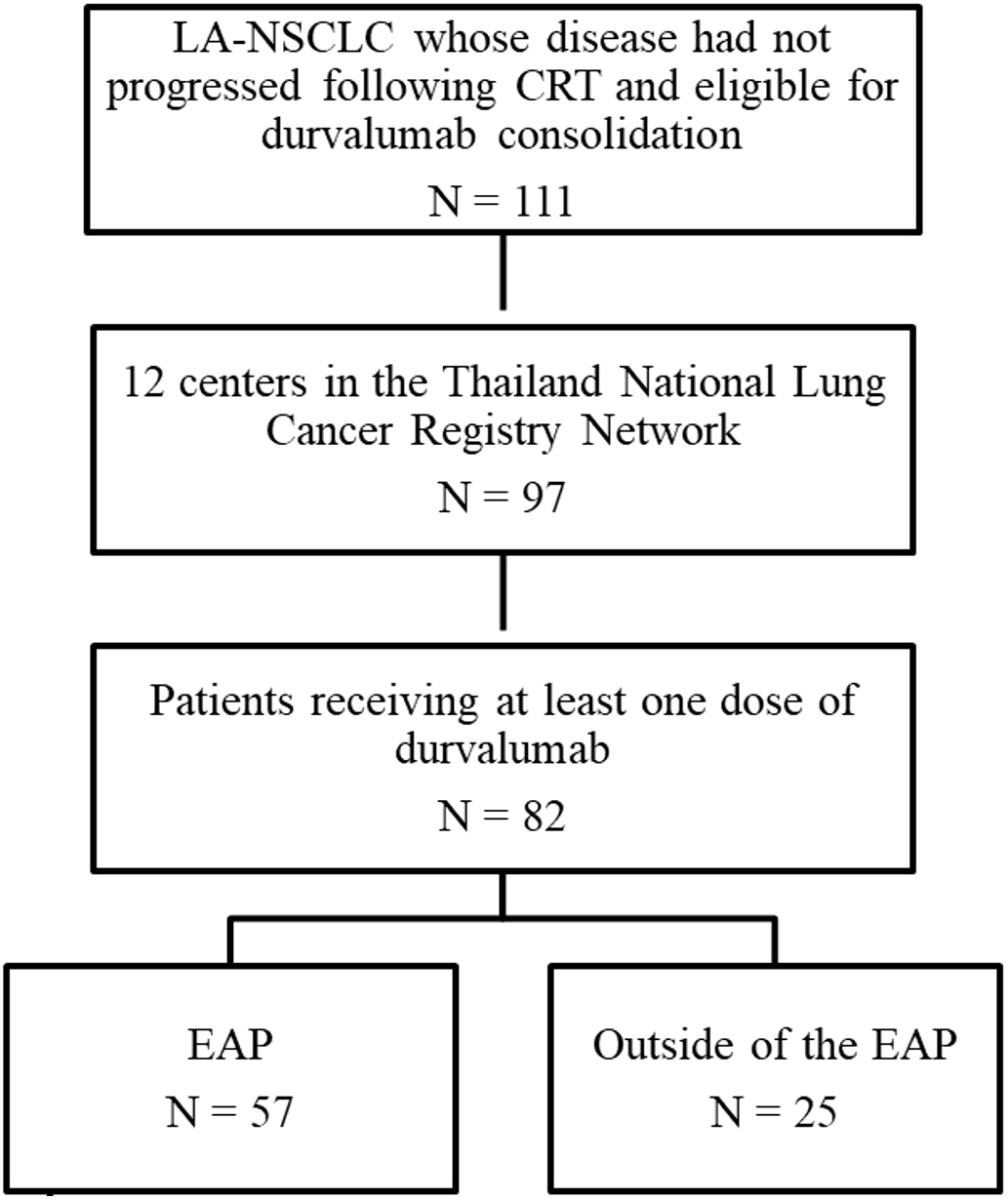
Flow diagram of the study.

A total of 111 patients diagnosed with LA-NSCLC, who had not experienced disease progression following concurrent platinum-based CRT, were included in this study. Among them, 82 patients were enrolled from 12 centers participating in the Thailand National Lung Cancer Registry Network (**Figure 1**). Baseline characteristics are summarized in **Table 1**. The median age of patients at the time of diagnosis was 63 years. Most were male (74%), exhibited Eastern Cooperative Oncology Group (ECOG) performance status (PS) of 0 to 1 (97%), and were current or former smokers (66%). At the initial diagnosis of NSCLC, most patients (89%) presented with stage III disease, while the remaining individuals experienced a relapse to stage III from earlier disease stages. In terms of disease stage, 9.8%, 31.7%, 36.6%, and 22% of patients had stages IA–IIB, IIIA, and IIIB or IIIC, respectively. Non-squamous NSCLC accounted for 72% of the cases. Forty patients (49%) underwent evaluation for *EGFR* mutation, and 26 patients (32%) were assessed for anaplastic lymphoma kinase (ALK) alterations. Within this subgroup, *EGFR* mutation and ALK alterations were observed in eight patients (20%) and three patients (11.5%), respectively. Only 13.4% of patients had undergone testing for programmed death-ligand 1 (PD-L1) expression, and 54.5% exhibited a PD-L1 tumor proportion score (TPS) of ≥1%. The majority of patients (84%) received concurrent CRT (cCRT), with carboplatin/paclitaxel being the most commonly administered regimen. The median total radiation dose administered was 60 Gray (Gy) (range 45–81). Most patients (81.7%) received radiation doses consistent with the PACIFIC protocol, which recommended a range of 54 to 66 Gy. However, a small proportion of patients deviated from this range. Specifically, five patients (7%) received a lower radiation dose than the PACIFIC range, while eight patients (11.3%) received a higher radiation dose. The best ORR to CRT, calculated from the baseline imaging before CRT to the imaging conducted prior to the initiation of durvalumab, was 65.9%. Among these, 54 patients achieved a partial response (PR), while 22 patients demonstrated stable disease (SD). The response was not evaluable in six patients.

**Table 1:** Patient baseline characteristics.

Baseline characteristics	All (n = 82)	EAP (n = 57)	Non–EAP (n = 25)	*p-*Value
Age, years				0.09
Median (range)	63 (56.8–69)	64 (59–70)	59 (52–66)	
<65	47 (57.3)	29 (50.9)	18 (72)	
≥65	35 (42.7)	28 (49.1)	7 (28)	
Gender, n (%)				0.79
Male	61 (74.4)	43 (75.4)	18 (72)	
Female	21 (25.6)	14 (24.6)	7 (28)	
ECOG PS, n (%)^†^				1.00
0	25 (31.6)	18 (32.1)	7 (30.4)	
1	52 (65.8)	36 (64.3)	16 (69.6)	
2	2 (2.5)	2 (3.6)	0	
Unknown	3	1	2	
Smoking status, n (%)				0.60
Current/former	51 (66.2)	36 (64.3)	15 (71.4)	
Never	26 (33.8)	20 (35.7)	6 (28.6)	
Unknown	5	1	4	
Histology, n (%)				0.01*
Non-squamous	59 (72)	46 (80.7)	13 (52)	
Squamous	23 (28)	11 (19.3)	12 (48)	
Stage, n (%)^‡^				0.71
IIB	3 (3.7)	2 (3.5)	1 (4)	
IIIA	26 (31.7)	19 (33.3)	7 (28)	
IIIB	29 (35.4)	21 (36.8)	8 (32)	
IIIC	18 (22)	12 (21.1)	6 (24)	
Local recurrence	6 (7.3)	3 (5.3)	3 (12)	
PD-L1 status, n (%)				0.71
Not tested	71 (86.6)	50 (87.7)	21 (84)	
Tested	11 (13.4)	7 (12.3)	4 (16)	
<1%	5 (45.5)	4 (57.1)	1 (25)	
1%–49%	2 (18.2)	1 (14.3)	1 (25)	
≥50%	4 (36.3)	2 (28.6)	2 (50)	
*EGFR* mutation status, n (%)				0.03*
Not tested	42 (51.2)	31 (54.4)	11 (44)	
Tested	40 (48.8)	26 (45.6)	14 (56)	
Mutation	8 (20)	8 (30.8)	0	
Wild type	32 (80)	18 (69.2)	14 (100)	
ALK status, n (%)				1.00
Not tested	56 (68.3)	42 (73.7)	14 (56)	
Tested	26 (31.7)	15 (26.3)	11 (44)	
Rearranged	3 (11.5)	2 (13.3)	1 (9.1)	
Non-rearranged	23 (88.5)	13 (86.7)	10 (90.9)	
CRT fashion, n (%)				0.18
Sequential	12 (14.6)	10 (17.5)	2 (8)	
Concurrent	69 (84.1)	47 (82.5)	22 (88)	
RT alone	1 (1.2)	0	1 (4)	
CRT regimen, n (%)				0.02*
Platinum–etoposide	11 (13.4)	6 (10.5)	5 (20.8)	
Platinum–paclitaxel	60 (73.2)	47 (82.5)	13 (54.2)	
Platinum–pemetrexed	8 (9.8)	2 (3.5)	6 (25)	
Platinum–gemcitabine	1 (1.2)	1 (1.8)	0	
Cisplatin alone	1 (1.2)	1 (1.8)	0	
RT dose (Gy), n (%)				0.74
PACIFIC protocol	58 (81.7)	40 (80)	18 (85.7)	
Outside PACIFIC protocol	13 (18.3)	10 (20)	3 (14.3)	
Unknown	11	7	4	
RT technique, n (%)				0.09
3D-CRT	20 (24.4)	15 (26.3)	5 (20)	
VMAT	18 (22)	8 (14)	10 (40)	
IMRT	40 (48.8)	31 (54.4)	9 (36)	
SABR	2 (2.4)	1 (1.8)	1 (4)	
Others	2 (2.4)	2 (3.5)	0	

ALK, anaplastic lymphoma kinase; CRT, chemoradiotherapy; EAP, early access program; ECOG PS, Eastern Cooperative Oncology Group Performance Status; EGFR, epidermal growth factor receptor; IMRT, intensity-modulated radiotherapy; PD-L1, programmed cell death-ligand 1; SABR, stereotactic ablative radiotherapy; VMAT, volumetric modulated arc therapy; 3D-CRT, three-dimensional conformal radiotherapy.

^†^ECOG PS denotes the Eastern Cooperative Oncology Group (ECOG) scale of performance status (PS) (a performance status grade of 0 indicates asymptomatic, 1 restricted in strenuous activity but ambulatory, and 2 ambulatory and capable of all self-care but unable to carry out any work activities).

^‡^Clinical staging was performed according to the eighth edition of the American Joint Commission on Cancer; T = primary tumor; N = regional lymph node metastasis; M = distant metastasis staging system.

Baseline characteristics were well-balanced between the EAP and non-EAP cohorts, except for a higher proportion of squamous cell carcinoma histology and wild-type *EGFR* mutation in the non-EAP cohort (**Table 1**).

### Durvalumab treatment characteristics

Fifty-two percent of patients adhered to the PACIFIC protocol by initiating durvalumab treatment within 42 days after completing radiotherapy (RT). The median duration between the end of RT and the start of durvalumab was 41.5 days (range 1–215). However, it is noteworthy that a subset of patients deviated from this recommended timeframe. Specifically, 27 patients (33%) commenced immunotherapy more than 6 weeks but less than 12 weeks, while 12 patients (14.6%) initiated durvalumab treatment after 12 weeks had elapsed since the completion of RT. Among the 75 patients with evaluable disease, the ORR and DCR were 41.3% and 86.7%, respectively. This consisted of complete response in five patients (6.7%), PR in 26 patients (34.7%), SD in 34 patients (45.3%), and progressive disease (PD) in 10 patients (13.3%). The median DoR was 31.8 months [95% CI, 22.9 to not estimated (NE)]. As of the data cut-off on December 31, 2022, four out of 82 patients (4.9%) remained on durvalumab treatment. In total, 47 patients (57%) successfully completed the 12-month treatment course with a median of 24 cycles (range 1–26), and the median duration of treatment was 11.4 months (range 0–20.2). Disease progression was the most common reason for the discontinuation of durvalumab, accounting for 17 cases (20.7%), followed by immune-related adverse events (irAEs) leading to discontinuation in 10 cases (12.2%) (**Table 2**).

**Table 2:** Reason for discontinuation of durvalumab.

Reason for discontinuation of durvalumab	n (%)
Completion of the 12-month treatment	47 (57.3)
Disease progression	17 (20.7)
Adverse events	10 (12.2)
Other causes	2 (2.4)
Unknown	1 (1.2)
Lost to follow-up	1 (1.2)

### Survival outcomes

A *post-hoc* analysis was conducted, aligning the index date with the initiation of durvalumab as employed in the PACIFIC and PACIFIC-R studies. The median PFS for the overall study cohort was 40.1 months (95% CI, 27.7 to NE). The 1-year and 2-year PFS rates were recorded at 68.7% (95% CI, 58.2%–79.2%) and 52.9% (95% CI, 41.8%–64%), respectively (**Supplementary Figure S1**).

**Figure 2:**
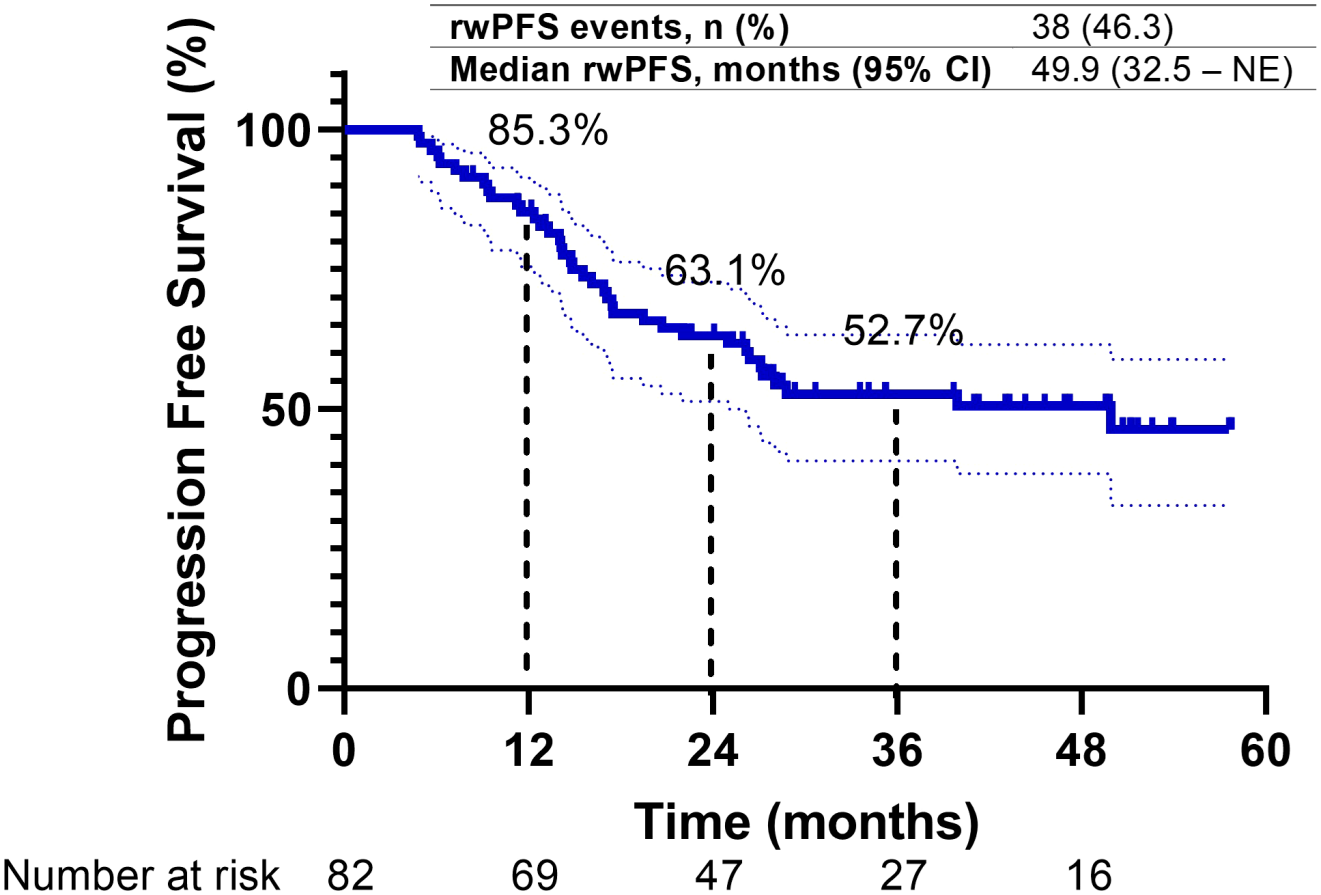
Progression-free survival of overall study cohort.

**Figure 3:**
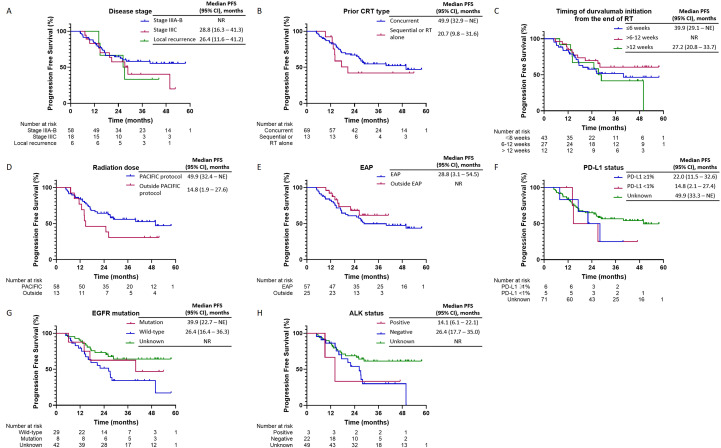
Progression-free survival in subgroups of interest. Kaplan-Meier curves depicting progression-free survival (PFS) over 60 months. Two groups are compared: patients without immune-related adverse events (irAEs) (blue line) and patients with irAEs (red line). The blue line indicates that median PFS was not reached (NR), while the red line shows a median PFS of 20.7 months (95% confidence interval [CI]: 2.2–39.1 months). The number of patients at risk decreases over time in both groups. **(A–H)** Progression Free Survival (PFS) stratified by clinical and molecular characteristics.

Upon a median follow-up period of 43.3 months (95% CI, 38.8–47.8) from diagnosis, disease progression was observed in 38 patients (46.3%), and 22 patients (26.8%) had died. The median PFS for the overall study cohort was 49.9 months (95% CI, 32.5 to NE). The 1-year and 2-year PFS rates were 85.3% (95% CI, 77.1%–93.5%) and 63.1% (95% CI, 52.4%–73.8%), respectively (**Figure 2**). PFS was longer in patients with a good PS (0–1) compared to those with a poor PS (≥2), and their median PFS was 49.9 and 26.2 months, respectively. Additionally, patients with stage IIIA/B disease exhibited longer median PFS (not reached) compared to those with stage IIIC disease (28.8 months) and local recurrence (26.4 months). Patients who received cCRT demonstrated a longer median PFS than those who received sequential CRT (sCRT) or RT alone, and their median PFS was 49.9 and 20.7 months, respectively. Moreover, patients who received radiation doses in accordance with the PACIFIC protocol exhibited an extended median PFS (49.9 months) compared to those who were outside the PACIFIC protocol (14.8 months). In contrast, the timing of durvalumab initiation from the end of RT did not significantly impact PFS. Patients who initiated treatment within 6 weeks, as recommended by the PACIFIC protocol, demonstrated a median PFS of 39.9 months. Those who initiated treatment beyond 6 to 12 weeks exhibited a median PFS that was not reached, while patients who initiated treatment after more than 12 weeks from RT had a median PFS of 27.2 months. Similarly, no substantial difference in PFS was observed between patients enrolled in the EAP and those treated outside of the EAP, and their median PFS was 28.8 months and not reached, respectively. Regarding PD-L1 expression, patients with a PD-L1 TPS of ≥1% had a median PFS of 22 months (95% CI, 11.5–32.6), while those with a TPS < 1% had a median PFS of 14.8 months (95% CI, 2.1–27.4) (*p* = 0.71). Although no statistically significant differences in PFS were detected between patients with *EGFR* mutations (EGFRm) and wild-type *EGFR* (EGFRwt), the median PFS was 39.9 months (95% CI, 22.7 to NE) for EGFRm and 26.4 months (95% CI, 16.4–36.3) for EGFRwt (*p* = 0.32). Conversely, patients with ALK rearrangement exhibited shorter PFS compared to those without ALK rearrangement, and their median PFS was 14.1 and 26.4 months, respectively (**Figures 3A–H**) (**Table 3**).

**Table 3:** PFS outcomes in the subgroup of interest.

Variables	Median PFS, months (95% CI)	PFS rate, %		*p-*Value
		1-year	2-year	
Age				0.45
<65	NR	85.1	60.7	
≥65	28.1 (9.5–46.6)	85.5	66.6	
Gender				0.24
Male	28.8 (8.9–48.6)	83.5	60.1	
Female	NR	90.5	71.4	
ECOG PS^†^				0.90
0–1	49.9 (32.4 to NE)	84.3	62.4	
≥2	26.2 (24.4 to NE)	100	50	
Smoking				0.36
Current/former	39.9 (15.6–64.3)	80.2	58.8	
Never	NR	92.3	73.1	
Histology				0.78
Non-squamous	39.9 (31.1 to NE)	81.3	60.6	
Squamous	49.9 (27.9 to NE)	95.7	69.8	
Stage^‡^				
IIIA–B	NR	83.6	62.8	
IIIC	28.8 (16.3–41.3)	83.0	57.4	
Local recurrence	26.4 (11.6–41.2)	83.3	66.7	0.47
CRT fashion				0.39
Sequential or RT alone	20.7 (9.8–31.6)	92.3	42.0	
Concurrent	49.9 (32.9 to NE)	83.9	67.0	
Radiation dose				0.11
PACIFIC protocol	49.9 (32.4 to NE)	84.5	64.4	
Outside PACIFIC protocol	14.8 (1.9–27.6)	76.9	46.2	
Time of durvalumab initiation				0.38
>12 weeks	27.2 (20.8–33.7)	91.7	66.7	
>6–12 weeks	NR	85.2	69.7	
≤6 weeks	39.9 (29.1 to NE)	83.6	57.8	
Time of durvalumab initiation				0.54
>14 days	49.9 (32.7 to NE)	84.4	63.3	
≤14 days	26.2 (6.2–46.1)	90.9	61.4	
PD-L1 status				0.45
≥1%	22.0 (11.5–32.6)	83.3	50	
<1%	14.8 (2.1–27.4)	100	50	
Unknown	49.9 (33.3 to NE)	84.4	65.0	
*EGFR* mutation status				0.32
EGFRm	39.9 (22.7 to NE)	87.5	62.5	
EGFRwt*	26.4 (16.4–36.3)	79	51.4	
Unknown	NR	90.5	73.1	
ALK status				0.66
Rearranged	14.1 (6.1–22.1)	66.7	33.3	
Non-rearranged**	26.4 (17.7–35.0)	86.7	51.0	
Unknown	NR	85.7	69.1	
Cohort				0.34
EAP	28.8 (3.1–54.5)	82.3	60.9	
Non-EAP	NR	92.0	68.4	

ALK, anaplastic lymphoma kinase; CRT, chemoradiotherapy; EAP, early access program; ECOG PS, Eastern Cooperative Oncology Group Performance Status; EGFR, epidermal growth factor receptor; EGFRm, epidermal growth factor receptor mutation; EGFRwt, epidermal growth factor receptor wild-type mutation; NE, not estimated; NR, not reached; PD-L1, programmed cell death-ligand 1; PFS, progression-free survival.

*EGFRwt represents patients whose test results were wild-type mutation and whose ALK results were negative or unknown.

**Non-rearranged ALK represents patients whose ALK results were negative and whose wild-type *EGFR* mutation was unknown.

^†^ECOG PS denotes the Eastern Cooperative Oncology Group (ECOG) scale of performance status (PS) (a performance status grade of 0 indicates asymptomatic, 1 restricted in strenuous activity but ambulatory, and 2 ambulatory and capable of all self-care but unable to carry out any work activities).

^‡^Clinical staging was performed according to the eighth edition of the American Joint Commission on Cancer (AJCC); primary tumor regional lymph node metastasis distant metastasis (TNM) staging system.

Additionally, the median PFS2 was 51.9 months (95% CI, 31.5–72.2), with corresponding 1- and 2-year PFS2 rates of 88.5% and 72.2%, respectively (**Supplementary Figure S2**). The interim analysis of median OS was not reached, and the 1- and 2-year OS rates were 89.7% and 75.1%, respectively (**Supplementary Figure S3**).

**Figure 4:**
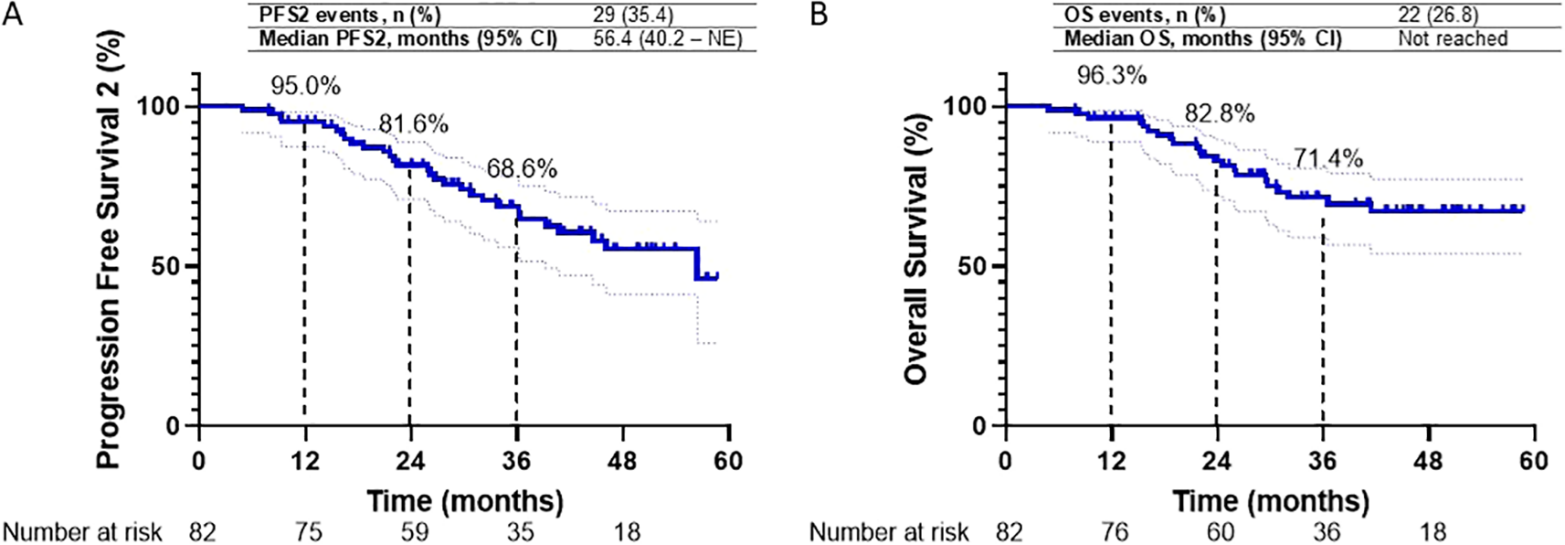
**(A, B)** Kaplan-Meier survival curves for Progression Free Survival2 (PFS2) and Overall Survival (OS) in the overall study cohort are presented. Graph A depicts PFS2 over 60 months, with 1-, 2-, and 3-year PFS2 rates of 95.0%, 81.6%, and 68.6%, respectively. Graph B illustrates OS over the same period, with 1-, 2-, and 3-year OS rates of 96.3%, 82.8%, and 71.4%, respectively.

As of the data cut-off date, a second progression event or death (PFS2) occurred in 29 patients (35.4%). The median PFS2 was 56.4 months (95% CI, 40.2 to NE), with 1- and 2-year PFS2 rates of 95.0% and 81.6%, respectively (**Figure 4A**). Interim analysis of median OS was not reached, with 1- and 2-year OS rates of 96.3% and 82.8%, respectively (**Figure 4B**).

A *post-hoc* analysis was conducted, aligning the index date with the initiation of durvalumab as employed in the PACIFIC and PACIFIC-R studies. The median PFS for the overall study cohort was 40.1 months (95% CI, 27.7 to NE). The 1-year and 2-year PFS rates were recorded at 68.7% and 52.9%, respectively (**Supplementary Figure S1**). Additionally, the median PFS2 was 51.9 months (95% CI, 31.5–72.2), with corresponding 1- and 2-year PFS2 rates of 88.5% and 72.2%, respectively (**Supplementary Figure S2**). The interim analysis of median OS was not reached, and the 1- and 2-year OS rates were 89.7% and 75.1%, respectively (**Supplementary Figure S3**).

### Pattern of recurrence

Thirty-eight patients experienced disease recurrence (46.3%). The initial recurrence pattern was characterized by locoregional relapse (LRR) in 12 patients (31.6%), distant relapse in 18 patients (47.4%), a combination of LRR and distant relapse in four patients (10.5%), and death in four patients (10.5%). The most common sites of distant relapse were the lung (n = 9, 23.7%), pleura (n = 7, 18.4%), and brain (n = 6, 15.8%) (**Supplementary Table S1**). Following the discontinuation of durvalumab treatment, subsequent therapy was administered to 29 patients (35.4%). Among them, 15 patients received chemotherapy, while five patients received targeted therapy. Furthermore, nine patients underwent subsequent radiotherapy (**Supplementary Table S2**).

### Safety

**Figure 5:**
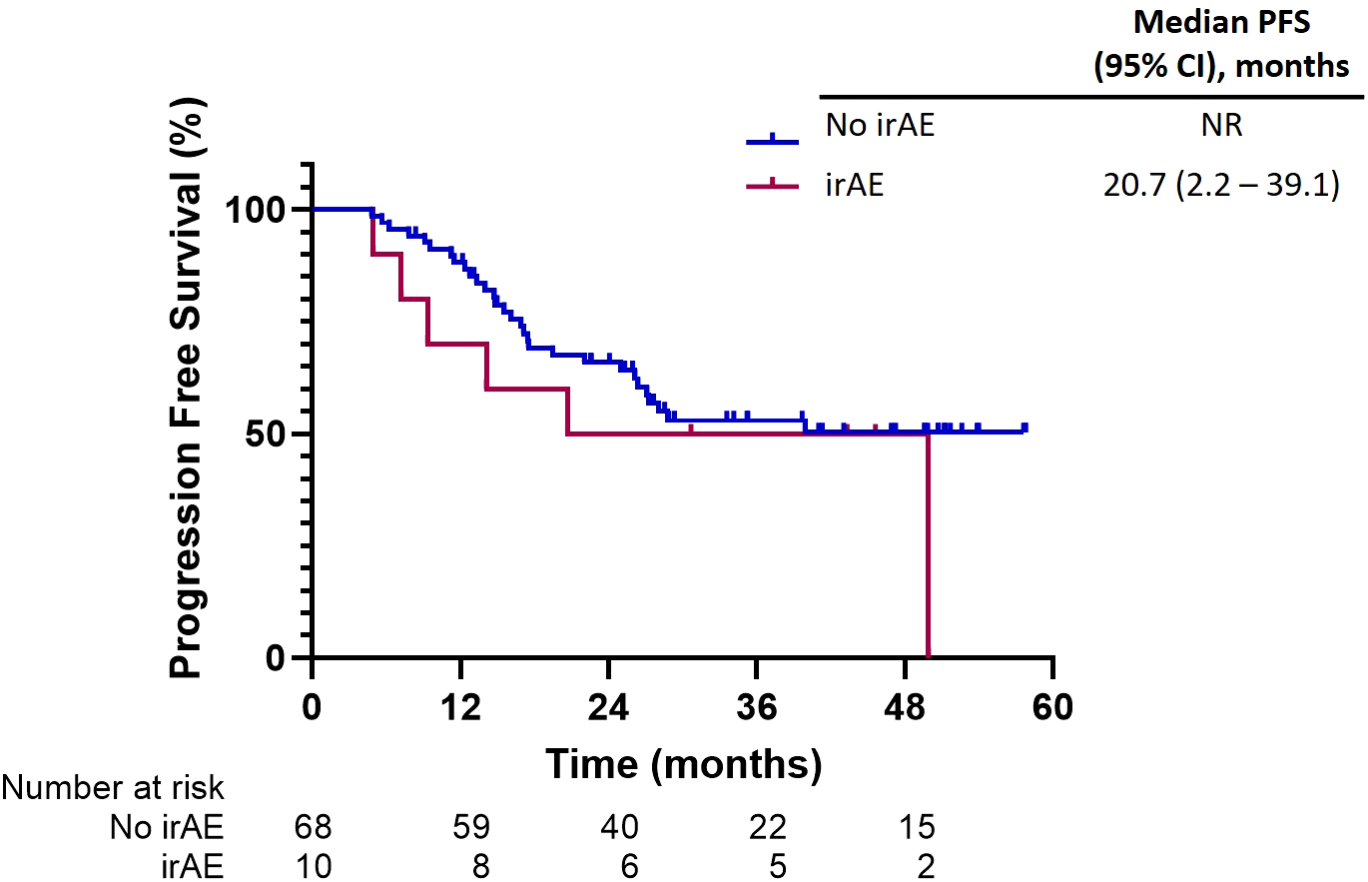
Progression-free survival among patients who discontinued treatment due to immune-related adverse events (irAEs).

Adverse events related to immune-related toxicities were documented in a significant proportion of patients in our study. Out of the total patient cohort, 21 patients (25.6%) experienced irAEs of any grade, while eight patients (9.8%) encountered irAEs of grade 3 or higher severity. Pneumonitis emerged as the most prevalent irAE, affecting 14 patients (17%), with five cases (6%) reaching grade 3 or higher. Consequently, the occurrence of pneumonitis necessitated treatment discontinuation for six patients (7.3%), and one patient (1.2%) experienced fatal pneumonitis events. However, before initiating durvalumab treatment, 14 patients had already developed radiation pneumonitis. Among them, five patients subsequently developed pneumonitis. Additionally, other commonly reported irAEs included hepatitis or transaminase elevation (9.8%) and hypothyroidism (8.5%). These adverse events led to the permanent discontinuation of treatment in 2.4% and 0% of patients, respectively (**Table 4**). PFS among patients who discontinued treatment due to irAEs exhibited outcomes consistent with the overall study population. The median PFS in this subgroup was 20.7 months (95% CI, 2.2–39.1). Furthermore, the 1-year and 2-year PFS rates were 88% and 65.9%, respectively (**Figure 5**).

**Table 4:** Immune-related adverse events (irAEs).

	All grades, n (%)	Grade ≥ 3, n (%)
Any irAEs	21 (25.6)	8 (9.8)
Skin dermatitis or rash	4 (4.9)	0
Pneumonitis or ILD	14 (17.1)	5 (6.1)
Diarrhea or colitis	2 (2.4)	0
Hepatitis or transaminase elevation	8 (9.8)	2 (2.4)
Pancreatitis or amylase/lipase elevation	1 (1.2)	0
Hypothyroidism	7 (8.5)	0
Hyperthyroidism	2 (2.4)	0
Nephritis or creatinine elevation	2 (2.4)	1 (1.2)
Infusion reactions	3 (3.7)	0
Neurological toxicities	2 (2.4)	0

ILD, interstitial lung disease.

## Discussion

This real-world, multicenter study of 82 patients represents the first investigation demonstrating the efficacy and safety of durvalumab as consolidation therapy following definitive CRT for patients with LA-NSCLC in Thailand, primarily focusing on Asian populations where the prevalence of *EGFR* mutation is higher compared to that in European populations. Patients with EGFR mutations were included in real-world studies of durvalumab as consolidation therapy for locally advanced NSCLC, with reported frequencies varying by geographic region: 26.2% in Taiwan (Wang 2021) (11), 10.3% in Japan (Tsukita 2021) (12), 9.5% in South Korea (Jung 2020) (13), 2% in the USA (Offin 2020) (14), and 1.8% in France (Avrillon 2021) (15). Our study identified EGFR mutations in 20% of patients. This proportion was similar to that reported in the Taiwan study.

With a median follow-up of 43.3 months from diagnosis, the 2-year PFS was 63.1%. From durvalumab initiation, the 2-year PFS was 52.9%. Despite considerable differences from the phase III PACIFIC trial regarding baseline characteristics and treatment strategies, our real-world cohort demonstrated meaningful clinical outcomes.

The median follow-up was 43.3 months (95% CI, 38.8–47.8) using the reverse Kaplan–Meier method. This duration substantially exceeded the 20-month enrollment period, reflecting the methodological distinction between enrollment timing and individual patient follow-up assessment. The reverse Kaplan–Meier approach estimates the theoretical follow-up duration that would have been achieved in the absence of clinical events, effectively projecting beyond the actual calendar observation period to provide a clinically meaningful measure of data maturity.

This analytical framework addressed two fundamental considerations in our study. First, it accounted for the temporal distinction between study-level enrollment windows and patient-specific follow-up trajectories, where early-enrolled patients had substantially longer observation periods than the enrollment window duration. Second, it provided an unbiased follow-up estimate that was independent of event occurrence, ensuring that survival analyses were based on adequate data maturity rather than being confounded by differential event rates across patient subgroups.

Patient populations differed substantially between EAP and non-EAP cohorts. EAP patients demonstrated inferior baseline characteristics, including poorer performance status, higher prevalence of stage IIIB/C disease, and greater use of sequential rather than concurrent chemoradiotherapy. Additionally, EAP patients experienced longer delays between radiotherapy completion and durvalumab initiation, often receiving suboptimal radiation doses. These differences reflected the inherent selection bias in EAP populations, which typically include patients with more advanced disease and broader eligibility criteria than those enrolled in pivotal trials (13, 16). Notably, 55.3% of our real-world cohort did not meet strict PACIFIC study inclusion criteria, highlighting the clinical reality of expanded access programs. Stratification by access program type is, therefore, methodologically essential to control for selection bias, lead-time bias, and confounding by indication, enabling differentiation between population-driven and treatment-driven outcome differences.

Our primary analysis from diagnosis demonstrated the real-world effectiveness of the complete treatment paradigm for LA stage III NSCLC in Thailand. The 2-year PFS of 63.1% from diagnosis compared favorably with historical outcomes and reflected the combined benefit of chemoradiation followed by durvalumab consolidation. *Post-hoc* analysis from durvalumab initiation (1-year PFS of 68.7% and 2-year PFS of 52.9%) enabled direct comparison with real-world data from pooled analysis demonstrating a 1-year PFS of 60% (95% CI, 56%–64%) (17). In addition, for subgroup analysis from this pooled analysis, the 1-year PFS was 61% (95% CI, 56%–65%) for Western population studies and 58% (95% CI, 47%–68%) for Asian population studies (17).

Our findings corroborated the efficacy of durvalumab as a consolidation therapy in a real-world clinical setting. Notably, our 1-year (68.7%) and 2-year PFS rates (52.9%) were higher than those reported in the PACIFIC study (55.7% and 45%, respectively) (18). Similar findings have been reported in other real-world studies from Western countries (10, 17, 19, 20), such as the PACIFIC-R study, which reported 1-year and 2-year PFS rates of 62.2% and 48.2%, respectively (10). However, the 1-year PFS rate observed in our study was considerably higher than that reported for the Asian population in the meta-analysis (68.7% when indexed from durvalumab initiation versus 58%, respectively) (17). This marked divergence from pooled Asian data warranted critical examination, particularly given the inherent methodological limitations of our single-country observational study. The superior outcomes observed in our relatively small cohort of 82 patients may reflect significant selection bias, as this sample likely represented only a fraction of the broader eligible population who received durvalumab consolidation therapy during the study period. Such discrepancies between institutional series and population-based analysis often indicate systematic differences in patient selection, treatment protocols, or outcome assessment methodologies that limit the generalizability of favorable results.

Additionally, there were no statistically significant differences in PFS between patients with PD-L1 TPS <1% and ≥1%. However, most patients in our cohort did not undergo PD-L1 expression testing; only 13.4% underwent PD-L1 testing, and 45.5% of those patients showed PD-L1 < 1%. Furthermore, our findings underscored the efficacy of durvalumab consolidation irrespective of the *EGFR* mutation status, which remained uncertain, as only 6% and 7.9% of patients in the PACIFIC and PACIFIC-R studies, respectively, had *EGFR* mutations (5, 10). Our study observed a significant prevalence of *EGFR* mutation and ALK alteration, recorded at 20% and 11.5%, respectively. Moreover, our cohort comprised a high proportion of individuals who had never smoked—33.8%. This contrasts sharply with the PACIFIC and PACIFIC-R studies, which reported only 9% and 7.9% of non-smokers, respectively.

Progression-free survival benefit was observed across all biomarker subgroups in our study, including patients with PD-L1 < 1%, EGFR mutations, and ALK rearrangement populations that typically exhibit limited response to immunotherapy in advanced disease. This biomarker-independent efficacy is particularly noteworthy given that EGFR mutation and ALK rearrangement tumors are traditionally classified as “immune-cold”, suggesting distinct mechanisms of immune activation within the post-chemoradiation microenvironment. Radiation-induced immunogenic cell death, antigen release, and microenvironment remodeling may overcome the inherent immunosuppressive characteristics of oncogene-driven malignancies.

However, with only eight EGFR-mutated patients and the small number of patients with PD-L1 testing (13.4%), this precluded meaningful sensitivity analysis, introducing selection bias that may not reflect the broader population of stage III NSCLC patients receiving durvalumab consolidation. These results represented a major limitation reflecting the lack of routine PD-L1 testing in stage III NSCLC during the study period. Pre-planned exploratory analysis was conducted to generate a hypothesis due to treatment effects across clinically relevant subgroups. These analyses were not powered for definitive conclusions regarding limited sample sizes, particularly for biomarker-defined subgroups. Validation in adequately powered prospective studies is required.

The discrepancy between real-world data and randomized controlled trials (RCTs) may be attributed to the use of retrospective data, which could introduce selection bias and data inconsistency issues. Additionally, disease progression assessments in real-world settings may occur less frequently or consistently compared to clinical trials, especially during the COVID-19 pandemic, potentially leading to an overestimation of PFS outcomes due to fewer hospital visits. Furthermore, since our study included patients treated in a single country, it increased data and clinical consistency but may limit generalizability to treatment approaches in other countries, given the heterogeneity in therapeutic approaches for LA-NSCLC. Moreover, our cohort did not include the assessment of other genetic alterations that may contribute to immunotherapy response.

In terms of safety, durvalumab treatment was well-tolerated in our real-world setting, with safety profiles consistent with those reported in the PACIFIC study. Over half of the patients in our study completed the 12-month treatment with a median of 24 cycles, confirming that durvalumab was generally well-tolerated, consistent with clinical trial findings. Pneumonitis or interstitial lung disease (ILD) was the most common irAE in our study. In comparison to the PACIFIC trial, our study registered a lower incidence of all-grade pneumonitis (17% in our cohort versus 33.9% in PACIFIC). However, our data indicated a higher prevalence of grade ≥ 3 pneumonitis (6% in our analysis versus 4.2% in PACIFIC) and a more frequent rate of treatment discontinuation due to pneumonitis (7.3% in our study versus 4.8% in PACIFIC). We also observed a higher rate of pneumonitis in patients who had previously experienced radiation pneumonitis (35.7% of patients), which aligned with other real-world data in Asian populations (12, 17, 19). However, with appropriate management, including corticosteroids in 85.7% of cases, complete resolution was achieved in 78.6% of patients. In contrast, Western studies reported a lower incidence of pneumonitis. A pooled meta-analysis encompassing 20 real-world studies revealed an incidence of all-grade pneumonitis at 21% (95% CI, 12%–30%) in Western populations, whereas studies focusing on Asian populations recorded a notably higher incidence of 47% (95% CI, 23%–70%) (17).

The higher grade ≥ 3 pneumonitis rate in our study compared to the PACIFIC study may reflect genetic predisposition, environmental factors, or healthcare practice differences in Asia. Our study found that the strong association with prior radiation pneumonitis suggested that heightened vigilance is required in this subgroup. Therefore, a comprehensive monitoring approach and exploring prophylactic strategies in high-risk subgroups are required for Asian patients. Furthermore, genetic biomarkers for pneumonitis prediction in Asian patients should be further investigated (21–23).

Several limitations should be acknowledged in our study. The most significant limitation stems from the inherent selection bias characteristic of single-country observational studies, representing a highly selected subset of the broader eligible patients who received durvalumab consolidation during the study period. Our study suffered from substantial gaps in biomarker characterization that limited the validity of subgroup analyses. This incomplete biomarker profiling introduced significant uncertainty into the subgroup analyses. Regarding the retrospective study design, the assessment of disease progression introduced potential lead-time and surveillance biases. The study remained underpowered for meaningful subgroup analyses. The small sample sizes within biomarker-defined subgroups precluded definitive conclusions about treatment efficacy. Furthermore, the single-country design significantly limited external validity. Thailand’s healthcare infrastructure, treatment protocols, and patient characteristics may not be representative of other Asian countries or healthcare systems. Moreover, the high proportion of patients treated within the expanded access program (69.5%) further limited generalizability to standard clinical practice settings.

## Conclusions

This real-world, multicenter study of Thai patients with locally advanced NSCLC demonstrates that durvalumab consolidation therapy following definitive chemoradiotherapy achieved favorable clinical outcomes consistent with the PACIFIC trial, despite including diverse patient populations with EGFR mutations and suboptimal characteristics typically underrepresented in registration trials. Durvalumab was generally well-tolerated; however, pneumonitis rates were higher than those in Western populations, emphasizing the need for enhanced monitoring in Asian patients. Although selection bias and incomplete biomarker characterization limited generalizability, these findings validated durvalumab consolidation as an effective standard of care in Asian populations with locally advanced NSCLC and underscored the need for larger prospective studies to definitively characterize treatment efficacy in biomarker-defined subgroups and develop predictive models for immune-related adverse events in Asian populations.

## Data Availability

The original contributions presented in the study are included in the article/**Supplementary Material**. Further inquiries can be directed to the corresponding author.
