# Supplementary material for: Durvalumab following definitive chemoradiotherapy among patients with stage III NSCLC in the Thai population: a real-world, multicenter observational study
Source: Front Oncol. 2025 Oct 22;15:1647385. doi: 10.3389/fonc.2025.1647385 (PMC12586107; doi:10.3389/fonc.2025.1647385)
Supplement: Supplementary file 1 [file DataSheet1.docx]

**Supplementary Information**

**Appendix.** Full protocol

**Supplementary Fig. S1.** Progression-free survival of the overall study cohort. (index date was the date of durvalumab initiation)

**Supplementary Fig. S2.** Progression-free survival 2 of the overall study cohort. (index date was the date of durvalumab initiation)

**Supplementary Fig. S3.** Overall survival of the overall study cohort. (index date was the date of durvalumab initiation)

**Supplementary Table S1.** Patterns of Disease Progression

**Supplementary Table S2.** Post-discontinuation Anticancer Therapy

Supplementary Fig. S1.

Supplementary Fig. S2.

Supplementary Fig. S3.

Supplementary Table S1.

| **Any progression, n (%)** | **N = 38 (46.3)** |
| --- | --- |
| Locoregional relapse | 12 (31.6) |
| Distant relapse | 18 (47.4) |
| Both | 4 (10.5) |
| Unknown or death | 4 (10.5) |
| **Any sites of distant relapse, n (%)** |  |
| Lung | 9 (23.7) |
| Pleura | 7 (18.4) |
| Brain | 6 (15.8) |
| Liver | 3 (7.9) |
| Bone | 2 (5.3) |
| Adrenal glands | 2 (5.3) |
| Others | 4 (10.5) |

Supplementary Table S2.

| **Any therapy, n (%)** | **N = 29 (35.4)** |
| --- | --- |
| Cytotoxic Chemotherapy | 15 (51.7) |
| Targeted Therapy | 5 (17.2) |
| Immunotherapy | 0 |
| Radiotherapy | 9 (31.0) |
| Death | 7 |
| Lost to follow-up | 7 |
